# Stressed Speech Emotion Recognition Using Teager Energy and Spectral Feature Fusion with Feature Optimization

**DOI:** 10.1155/2023/5765760

**Published:** 2023-10-11

**Authors:** Surekha Reddy Bandela, S. Siva Priyanka, K. Sunil Kumar, Y. Vijay Bhaskar Reddy, Afework Aemro Berhanu

**Affiliations:** ^1^Department of ECE, Institute of Aeronautical Engineering, Hyderabad, India; ^2^Department of ECE, KITS, Warangal, Telangana, India; ^3^Research Scholar NIT, Warangal, Telangana, India; ^4^Laki Reddy Bali Reddy College of Engineering, Mylavaram, Andhra Pradesh, India; ^5^Department of Environmental Engineering, College of Biological and Chemical Engineering Addis Ababa Science and Technology University, Addis Ababa, Ethiopia

## Abstract

The objective of speech emotion recognition (SER) is to enhance man–machine interface. It can also be used to cover the physiological state of a person in critical situations. In recent time, speech emotion recognition also finds its operations in medicine and forensics. A new feature extraction technique using Teager energy operator (TEO) is proposed for the detection of stressed emotions as Teager energy-autocorrelation envelope (TEO-Auto-Env). TEO is basically designed for increasing the energies of the stressed speech signals whose energies are reduced during the speech production process and hence used in this analysis. A stressed speech emotion recognition (SSER) system is developed using TEO-Auto-Env and spectral feature combination for detecting the emotions. The spectral features considered are Mel-frequency cepstral coefficients (MFCC), linear prediction cepstral coefficients (LPCC), and relative spectra–perceptual linear prediction (RASTA-PLP). EMO-DB (German), EMOVO (Italian), IITKGP (Telugu), and EMA (English) databases are used in this analysis. The classification of the emotions is carried out using the k-nearest neighborhood (k-NN) classifier for gender-dependent (GD) and speaker-independent (SI) cases. The proposed SSER system provides improved accuracy compared to the existing ones. Average recall is used for performance evaluation. The highest classification accuracy is achieved using the feature combination of TEO-Auto-Env, MFCC, and LPCC features with 91.4% (SI), 91.4% (GD-male), and 93.1%(GD-female) for EMO-DB; 68.5% (SI), 68.5% (GD-male), and 74.6% (GD-female) for EMOVO; 90.6%(SI), 91% (GD-male), and 92.3% (GD-female) for EMA; and 95.1% (GD-female) for IITKGP female database.

## 1. Introduction

Speech emotion recognition (SER) is the task of recognizing the emotional aspects of speech irrespective of the semantic contents. Emotion recognition provides benefits to numerous institutions and aspects of life. It is useful and important for healthcare and security purposes. Also, it is vital for easy and simple detection of human feelings at a specific moment without actually asking them. Emotion extraction from speech, or speech emotion recognition, is the technique of identifying the speaker's emotional state (SER). The ability to recognize and interpret the speaker's emotional state is crucial for human–computer interaction. SER is used in a wide range of applications, including call centres, autos, medical services, e-tutoring and story-telling, and many more. SER can be used in a variety of ways. Depending on the context and the speaker, human speech encompasses a wide spectrum of emotions. Emotions can be categorized into six fundamental archetypal emotions based on their intensity [1, 2]. Anger, joy, surprise, contempt, fear, and sadness are types of emotions. If you are creating a speech recognition system, you need to take into account how the speaker's emotional state will be conveyed by extracting parts from their voice signal. In terms of speech elements that are influenced by emotions, we can classify them as follows: qualitative, spectral, continuous, and Teager energy operator-based features. The emotional content of a speech utterance has a significant impact on its pitch, zero crossing rate, and energy. Speech's vitality, articulation rate, spectral data, and basic frequency fit under this umbrella term (*f*_0_). Observed emotion and the quality of the voice are closely linked. The categories for these structures are voice level, pitch, temporal, and feature boundary structures. The spectral analysis of a voice signal yields a short temporal representation of the signal's characteristics. The emotional content of a spoken utterance determines the spectral energy distribution in the speech utterance. For instance, high-arousal emotions, such as gladness (or) wrath, are connected with high energies in the higher frequencies, while low-arousal emotions, such as melancholy, are associated with lower energies in the same frequency range. The nonlinear airflow in the vocal tract system is responsible for speaking. The speaker's vocal tract system, which is responsible for producing sound, is affected by the speaker's muscle tension when he or she is stressed. As a result, nonlinear speech features are essential for accurately identifying human speech in recorded audio. By using the Teager energy operator (TEO) technique, a feature fusion of the TEO-Auto-Env and spectral features using k-NN classifier for SSER system improves the accuracy when compared to the existing ones. The features that cannot be extracted by the existing ones can be extracted by combing TEO-Auto-Env and spectral features.

### 1.1. Related Work

Beginning in the early 1990s with the purpose of detecting displeasure or annoyance in the speaker's speech, the field of voice recognition has since grown to include a wide range of applications. A variety of real-time applications require the ability to detect stressed emotions in speech. These applications include preventing car accidents, providing appropriate counselling to students, and assisting children's parents and other close relationships by training speech recognition systems on stressed speech [3]. Many disasters can be averted if people are aware of their own high-stress emotions and can recognize them in advance. Until recently, the majority of research has focused on recognizing all of the distinct emotions, with little emphasis dedicated to the more severe ones, such as anger, fear, boredom, melancholy, disgust, and impatience, which can be painful.

So far, MFCC is the spectral feature providing promising results for speech emotion recognition. But for the depression detection, the main focus must be on the stressed emotions such as anger, sad, and so on. Hence, the stressed emotion detection was started with modification and feature fusion of these different speech features. For the stressed or depressed emotion recognition, the existing feature like MFCC was improved as modified MFCC (M-MFCC), ExpoLog based scale, LPC improvised as one-sided autocorrelation LPC (OSALPC) [4], and a new technique with feature fusion of MFCC and short time energy features with velocity (Δ) and acceleration (Δ + Δ) [5]. Feature extraction techniques were used for stressed emotion recognition whose performance was better compared to the existing MFCC and LPCC methods. Later, specifically for anger emotion recognition, acoustic (pitch, loudness, spectral features) and linguistic (probabilistic and entropy-based words and phrases) cues [6] were introduced. Apart from these, other different feature extraction techniques such as a sinusoidal model-based feature extraction technique with frequency, magnitude, and phase features [7]; empirical mode decomposition method; feature optimization method [8] to select particular frames of the speech signal by choosing proper filter bank; hybrid biogeography-based optimization; and particle swarm optimization (BBO_PSO) [9] by the proper selection of higher-order spectral features were used for depressed emotion recognition. Yang and Lugger [10] proposed the combination of qualitative and voice quality features; Wen et al. [11] proposed weighted spectral local Hu parameters to overcome the disadvantage of MFCC feature; Wang et al. [12] proposed Fourier parameters; Setayeshi et al. [13] proposed a bioinspired ANFIS technique combined with MLP for SER for anger, happy and sad emotions; and, Ying and Xue-Ying [14] proposed glottal compensation to zero crossings with maximal Teager energy operator (GCZCMT) for speech emotion recognition and performed well compared to MFCC feature. The Glottal Function Index (GFI) is a validated, reliable, and easily self-administered four-item battery that is aimed specifically at identifying the presence and degree of vocal cord dysfunction in adults. GCZCMT feature is a feature possibly and effectively distinguishing emotional state. It has high practical value and best suited for actual complex language environment. But few of the stressed emotions such as anger, disgust, sad, and so on, were not accurately detected using these features also.

Teager and Kaiser [15, 16] developed a feature called Teager energy operator (TEO) to recognize strained emotions for the first time based on the concept that hearing is the mechanism of energy detection. Emotional stress can be detected with the Teager energy operator (TEO). An energy profile-driven pitch contour [17] has been created for Lombard and fury emotion recognition based on this. Neutral and stressed speech can be distinguished using TEO-FM-Var, normalised autocorrelation envelope Area, and critical band autocorrelation envelope area [18, 19]. For clinically depressed or stressed speech detection, the TEO-CB-Auto-Env was employed in conjunction with several low-level descriptors (LLDs), such as pitch, formants, energy, and their delta and delta-delta as well as spectral features (spectral flux, entropy, and their centroid) and their delta. TEO-CB was found to exist. This procedure, however, is complicated because of the enormous number of factors involved. Multiple feature fusion techniques [20–22] combining glottal, prosodic, spectral, and TEO-based characteristics were later presented [20–22] for the detection of stressful emotions. [19] reported the influence that classification accuracies have in speech analysis from a clinical dataset by appending acoustic low-level descriptors(LLD) belonging to prosodic (i.e. pitch, formants, energy, jitter, shimmer) and spectral features (i.e. spectral flux, centroid, entropy, and roll-off) along with their delta(Δ) and delta-delta(Δ-Δ) coefficients to two the baseline features of Teager energy critical band-based autocorrelation envelope and Mel-frequency cepstral coefficients [23] collected and analyzed the movement data of the jaw, the tongue tip, and the lower lip, along with speech, and research differences in speech articulation among four emotion types: neutral, anger, happiness, and sadness. The effectiveness of the articulatory parameters in emotion classification was also investigated.

The collected characteristics were fed into a variety of classification models, including support vector machine (SVM), vector quantization (VQ), Gaussian mixture model (GMM), k-NN, and several neural network (NN) techniques [1, 24, 25].

The highest accuracy achieved by existing work is 91%, which is overcome by this proposed technique.

### 1.2. Speech Emotion Recognition System

**Figure 1:**
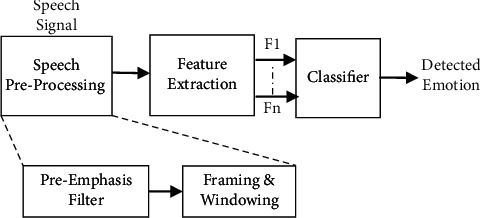
Speech emotion recognition system.

The preprocessing, feature extraction, and classifier blocks of the basic speech emotion recognition (SER) system are illustrated in Figure 1 as the system's building parts. Physical quantities in the speech signal are delivered to the feature extraction block after they have been processed in the preprocessing stage. The features *F*_1_, *F*_2_,…, *F*_*n*_ are presented to the classifier in this section. Finally, this classifier is capable of distinguishing between different emotional states.

Because of this preprocessing, the feature extraction module will be able to process the speech signal more quickly and more accurately. Prior to image processing, there are three steps of preprocessing: filter, framing, and window. After the preprocessing procedure, the speech signal is used to extract physical properties including pitch, energy, and formants [26]. Filtering reduces the amount of noise in a spoken signal either during the recording process or as a result of disruptions to the recording environment. As a way to boost the intensity of speech signals at higher frequencies, a preemphasis filter is used. Nonstationary signals, such as speech, are difficult to analyze since they are nonstationary by definition. In this way, the voice signal can be analyzed independently of one another because it is divided into an equal number of samples. The size of the frame is governed by the feature extraction method used. To avoid the mismatch between the frames, some overlap between the frames is permitted. When the incoming data signal is divided into segments, some discontinuities arise at the frame boundaries as a result (frames). This discontinuity is avoided by passing each frame via a tapered glass.

**Figure 2:**
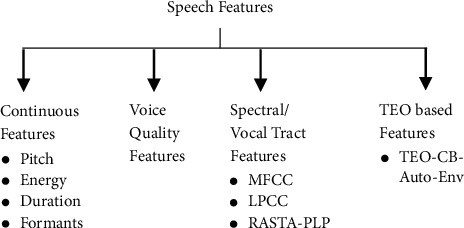
Categorization of speech features.

It is important to identify a specific speech feature extraction technique in speech emotion recognition that can classify the emotions from speech efficiently. So far, many speech features have been investigated for speech emotion, but the best speech features is not yet discovered.  Figure 2 shows some of the examples by categorizing the speech features [1, 2]. However, the combination of speech features to represent the speech signal is the most common practice in speech emotion recognition. In this work, a new TEO-based feature is proposed and is combined with the spectral features. Among these, the SSER system developed using the proposed feature fusion is compared with existing spectral features MFCC, LPCC, and RASTA-PLP-based SSER systems.

In this paper, a new feature based on TEO, i.e., TEO-Auto-Env is combined with MFCC, LPCC, and RASTA-PLP, and the combined features are further optimized using principal component analysis. Later, the emotions are classified using k-NN classifier in the proposed stressed speech emotion recognition system. The proposed methods provide better performance and are also simple to design compared to the previous existing methods.

The following is the structure of this paper: Section 2 covers the database that was used in this study, followed by Section 3, which discusses the proposed feature extraction approach that makes use of TEO.  Section 4 presents the simulation results of the proposed stressed speech emotion recognition system, which is based on feature fusion and optimization.  Section 5 concludes the study by summarizing the findings of the paper.

## 2. Database Description

The speech emotion database is also one of the challenges in the analysis of speech emotion recognition. The classification accuracy varies with different datasets. There is no standard database in all languages that is accepted by all the emotion recognition researchers so far. EMO-DB is the German database, i.e., mostly considered by many of the researchers and hence used in this paper. Apart from this EMOVO (Italian), IITKGP (Telugu), and EMA (English) databases are also considered. The description of the databases is discussed in the following sections.

### 2.1. EMO-DB Database

This is a Berlin Database of Emotional German Speech compiled by the University of Berlin. Five male and five female performers between the ages of 25 and 35 were recorded in an anechoic room for the purpose of capturing emotional data. In the chart, the emotions were labelled as follows: anger (1), boredom (2), disgust (3), fear (4), happiness (1), sad (2), neutral (1), and fear (3).  Table 1 shows the performers each delivered a total of 10 different phrases onstage. The following is a breakdown of the database distribution.

### 2.2. EMOVO Database

It is the first emotional database in Italian language [27]. The recordings were done in Fondazione Ugo Bordoni laboratories. Six actors with three male and three female speakers has spoken14 sentences in six different emotions, namely, disgust (D), fear (F), anger (A), joy (J), surprise (Su), sadness (S) apart from neutral (N) speech were recorded.  Table 2 shows the distribution of EMOVO Database.

Among these disgust, fear, anger, joy/happiness, sad, and neutral are considered for the analysis in this paper.

### 2.3. IITKGP Telugu Database

This is the first Telugu database created by IIT Kharagpur [28]. The recordings were done by radio artists with 15 sentences spoken by 10 different speakers. The emotions recorded were anger, compassion, disgust, fear, happy, neutral, sarcastic, and surprise. Among this, only five female speaker data consisting of anger, compassion, happy, and neutral are used in this analysis. The distribution of these emotions is as shown in Table 3.

### 2.4. EMA Database

This Electromagnetic Articulography (EMA) database [29] comprises of one male and two female native speakers of American English. A total of 14 sentences for male and 10 sentences for female speakers with five repetitions of each sentence in four different emotions, namely, neutral, anger, sad, and happiness were recorded. All the emotions are considered in this analysis. The distribution of these emotions is as shown in Table 4.

## 3. Stressed Speech Emotion Recognition (SSER) System Using the Feature Fusion of Proposed TEO-Auto-Env and Spectral Features

**Figure 3:**
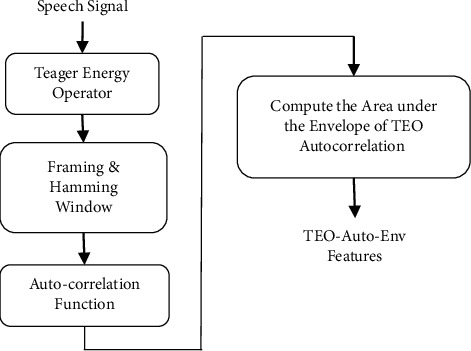
Teager energy autocorrelation envelope (TEO-Auto-Env) feature extraction.

The proposed stressed speech emotion recognition (SSER) system is as shown in Figure 3 to classify the stressed emotions such as anger, fear, disgust, sad, and so on, effectively compared to the existing methods. In this proposed system, the speech signal is given to the feature extraction block with the feature fusion of Teager energy-based feature, i.e., TEO-Auto-Env and spectral feature.

### 3.1. Teager Energy Autocorrelation Envelope (TEO-Auto-Env) Feature Extraction

The TEO-Auto-Env feature extraction is depicted in Figure 3.

#### 3.1.1. Teager Energy Operator (TEO)

**Figure 4:**
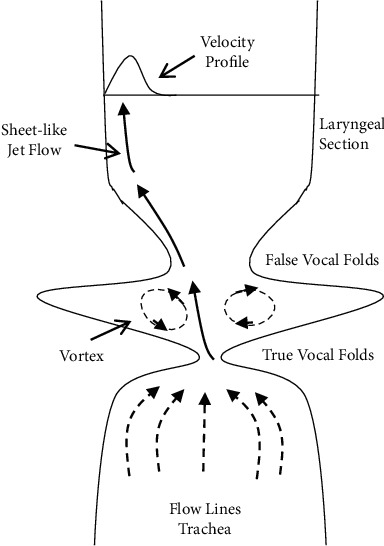
Nonlinear model of sound propagation along the vocal tract.

An energy operator, known as the Teager energy operator, was developed by Teager [17] as a measure of speech signal energy. Teager energy operator(TEO) substantially shows the frequencies and immediate changes of the signal amplitude that's truly sensitive to subtle changes. Although TEO was first proposed for modelling nonlinear speech signals, it was later extensively applied in the audio signal processing. It gives high reliability and accuracy. Teager's research showed that the flow of oxygen in the vocal tract is separated and follows the walls of the vocal tract. Based on the findings of a few whistle experiments illustrated in Figure 4, Teager presented the vocal tract geometry and modelled speech production. Figure 4 depicts the anatomy of the vocal tract and a speech production model. Here, air is expelled as an aerosol and adheres to the vocal tract wall closest to where the jet is escaping. Air vortices arise in the hollow as a result of the forced passage of air between the real and false vocal folds. There is a large amount of air that passes close to the lips during air's propagation as it follows the walls of the vocal tract. One of the most important aspects of this model is the vortex. An unconstructed vocal tract with sound produced just at the glottis is what is assumed in the traditional speech production concept [23]. However, Teager contends that the voice signal is modulated as a result of the active sound production of vortices around the artificial vocal folds.

On the other hand, Teager later conducted a series of studies on the hearing process and came up with a measurement of the energy parameter that could be used to prove that speech modulation patterns exist in the environment. This is the first time that Kaiser [16] has proven the energy operator in his study.

(1) $$\begin{array}{c} \text{TEO}\left(x\left(t\right)\right)={\left(\frac{d}{\text{d}t}x\left(t\right)\right)}^{2}-x\left(t\right)\left(\frac{d^{2}}{\text{d}t^{2}}x\left(t\right)\right), \end{array}$$

or

(2) $$\begin{array}{c} \text{TEO}\left(x\left[n\right]\right)=\;x^{2}\left[n\right]-x\left[n\right]x\left[n+1\right], \end{array}$$

where “*x*(*t*)” and *x*[*n*] are the continuous and discrete speech signals.

**Figure 5:**
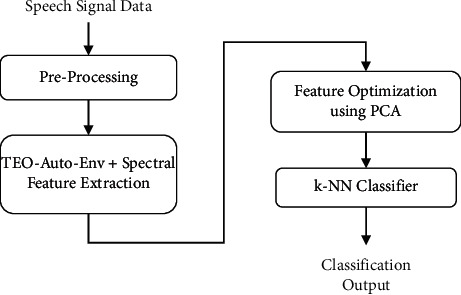
SSER system using the proposed feature combination of TEO-Auto-Env and spectral features.

When a voice signal is created under a stressful scenario, the nonlinear flow of air in the vocal tract system is disrupted, resulting in decreased vocal quality. When it comes to speech identification, nonlinear qualities like these are important to success. TEO is mostly used to discriminate between Lombard, furious, and loud emotions, as well as between neutral and negative emotions.  Figure 5 illustrates what I'm talking about: the voice signal is not preemphasis filtered, but it is routed through the TEO block before reaching the receiver [30]. When strained emotions pass over the TEO barrier, there is an increase in the amount of stress-related emotional energy released. In order to further magnify the supercharged voice stream, framing and windowing blocks are employed. These frames are taken into consideration by the autocorrelation function.

#### 3.1.2. Autocorrelation Function (ACF)

The ACF is a time-dependent correlation between a signal and a delayed copy of that same signal in signal processing. How comparable two observations are to each other as a function of how long it has been since they were made.

(3) $$\begin{array}{c} R_{xx}\left(k\right)=\sum_{n=k}^{M-1}s\left[n\right]s\left[n-k\right]. \end{array}$$

When the function gets a signal with the name “*x*(*t*),” the argument is used to delay the signal until the end of the function. It occurs when the autocorrelation function receives frames of the Teager-energized signal and applies the autocorrelation function to them. If the correlation between adjacent frames is particularly strong, the energy of the audio signal can be amplified even higher by detecting the correlation between adjacent frames. In order to proceed with further processing, the autocorrelated sequence is handed to the extraction block of the envelop.

#### 3.1.3. Area under the Envelope of TEO Autocorrelation

Thereafter, the area under the envelope of the TEO autocorrelation sequence obtained is calculated using trapezoidal numerical integration. The resultant values obtained are the Teager energy autocorrelation envelope (TEO-Auto-Env) features.

### 3.2. Spectral Features

**Figure 6:**
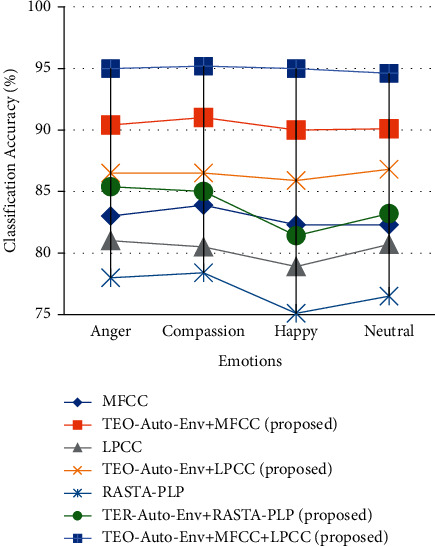
Comparisons of classification accuracies of SSER system for different emotions using the individual spectral features and the feature fusion of the proposed (TEO-Auto-Env) + spectral feature extraction techniques for IITKGP Telugu database for GD-female case.

Spectral features provided good accuracy so far in emotion classification. But, the drawback of these features is, they treat all the emotions similarly. But, for the stressed emotions, during the speech production process, the energy of the speech signal is deteriorated. Because of this, the complete features of these emotions were not perfectly extracted using the spectral features. Hence, there was a need of a feature to increase the energies of these stressed emotions [31]. A feature based on TEO can be used for this purpose. TEO-Auto-Env is combined with each of the spectral features, i.e., MFCC, LPCC, and RASTA-PLP to extract the features as shown in Figure 6. These combinations of features are given to the classifier to detect the emotions.

Therefore, a new feature based on TEO is proposed in this work, and this feature is combined with the spectral features in order to extract the features of the stressed emotions that were not effectively extracted by spectral features alone. The spectral features considered for the analysis are MFCC, LPCC, and RASTA-PLP.

#### 3.2.1. Spectral Features

(1) Mel-frequency cepstral coefficients (MFCC): most often used algorithms for spectrum transformations in voice recognition and emotional expression recognition are MFCCs. Using cepstral analysis, computers can imitate the human ear's perception. As mentioned in [32], in order to compute MFCCs, the speech must be divided into frames.

(2) Linear prediction cepstral coefficients (LPCC): in the vocal tract system, it is made up of a mixture of the excitation source as well as the time-varying components. To pinpoint the source and system components in the time domain so that they may be independently evaluated, linear prediction (LP) analysis is utilised.

(3) Cepstrum analysis: it is used to separate the source and system parameters of speech production process without the a priori information about source or system parameters. The cepstrum is defined as the inverse discrete Fourier transform (IDFT) of log magnitude of the DFT of a signal,

(4) $$\begin{array}{c} c\left[n\right]=\text{ID}FT\left\{\log\left|DFT\left\{x\left[n\right]\right\}\right|\right\}, \end{array}$$

where *x*[*n*] is the input signal.

(4) RASTA-PLP: it uses RASTA filtering in perceptual linear prediction (PLP). Perceptual processing, such as critical band analysis, equal loudness preemphasis, and intensity loudness, is performed before executing autoregressive (AR) modelling. PLP coefficients [33] are created from LP coefficients before performing AR modelling. RASTA filtering [34, 35], also known as bandpass filtering in the log spectral domain, was invented at the same time as PLP. By using this, the slow variations in the channel are suppressed. A general RASTA filter is defined by

(5) $$\begin{array}{c} T\left(z\right)=\frac{k\sum_{n=o}^{N}\left(n-\left(n-2\right)/2\right)z^{n}}{1-px^{-1}}, \end{array}$$

where the numerator is a regression filter of *N*th order and denominator is an integrator.

Each of these spectral features is combined with TEO-Auto-Env feature and is used for stressed speech emotion recognition.

### 3.3. Feature Optimization Using PCA

The curse of dimensionality can be alleviated before classifying emotions by selecting features through optimization. Training and classification performance suffers as a result of the higher-dimensional feature set's tendency to promote overfitting. Additional advantages include a reduction in the difficulty of collecting data, a boost in classifier unambiguity, and an increase in classification accuracy [36–38]. Following feature extraction, this work uses the principal component analysis (PCA) optimization technique to choose the most important characteristics and improve the classification algorithm's performance. If there are “*N*” measurements or samples in an n-dimensional space to be compressed, PCA searches for “*d*” n-dimensional orthonormal vectors that represent the data in a best manner, where *d* ≤ *n*.

#### 3.3.1. Principal Component Analysis (PCA)

Implementation steps of PCA:

- Take the whole dataset consisting of *d*-dimensional samples ignoring the class labels.
- Compute the *d*-dimensional mean vector (i.e., the means for every dimension of the whole dataset)
- Compute the scatter matrix (alternatively, the covariance matrix) of the whole data set.
- Compute eigenvectors (*e*_1_, *e*_2_, ..., *e*_*d*_) for the corresponding eigen values (*λ*_1_, *λ*_2_, ..., *λ*_*d*_)
- Sort the eigenvectors by decreasing order of eigenvalues and choose “*k*” eigenvectors with the largest eigenvalues to form a *d* × *k* dimensional matrix *W* (where every column represents an eigenvector)
- Use this *d* × *k* eigenvector matrix to transform the samples onto the new subspace. The resultant “*y*” is the transformed *k* × 1-dimensional sample in the new subspace.

### 3.4. Emotion Classification Using k-NN

The features selected using this PCA are used by the k-NN classifier for the classification of the emotions. Instead of finding the probability density function as in Gaussian mixture model, k-NN implicitly finds the decision boundaries of the different emotional features [39, 40]. The Euclidean distance measure algorithm is used to find the nearest neighbors to a specific emotional feature set boundaries. The training data are labelled accordingly with their emotional class labels. Given a point “*x*” to be classified, “*k*” nearest neighbors of “*x*” is selected and the point “*x*” is assigned to majority label of the “*k*” neighbors.

## 4. Simulation Results

**Figure 7:**
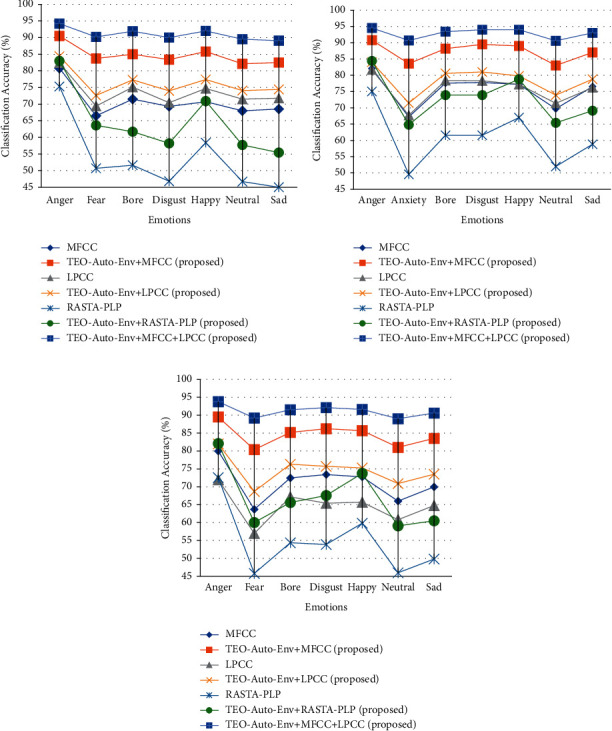
Comparisons of classification accuracies of SSER system for different emotions using the individual spectral features and the feature fusion of the proposed (TEO-Auto-Env) + spectral feature extraction techniques for EMO-DB German database for (a) GD-male, (b) GD-female, and (c) SI case.

**Figure 8:**
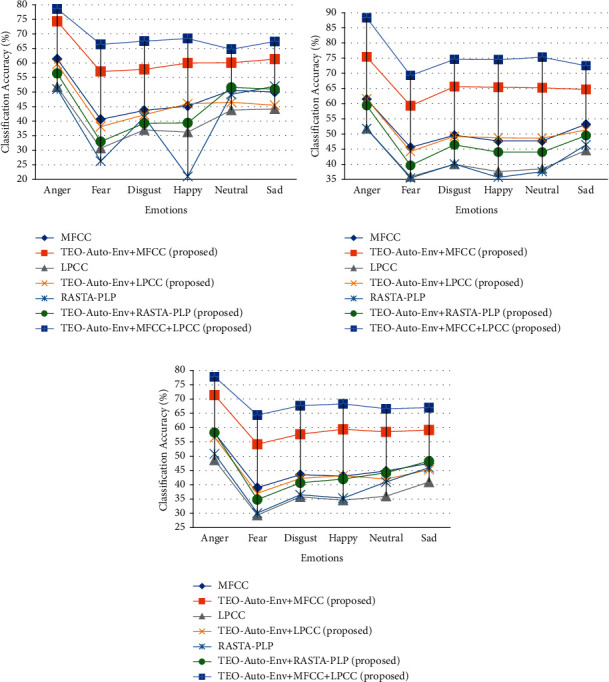
Comparisons of classification accuracies of SSER system for different emotions using the individual spectral features and the feature fusion of the proposed (TEO-Auto-Env) + spectral feature extraction techniques for EMOVO Italian database for (a) GD-male, (b) GD-female, and (c) SI case.

**Figure 9:**
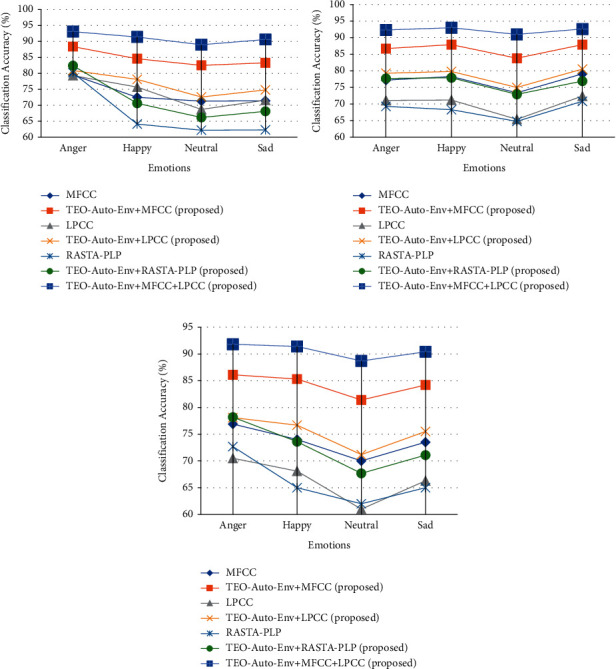
Comparisons of classification accuracies of SSER system for different emotions using the individual spectral features and the feature fusion of the proposed (TEO-Auto-Env) + spectral feature extraction techniques for EMA English database for (a) GD-male, (b) GD-female, and (c) SI case.

**Figure 10:**
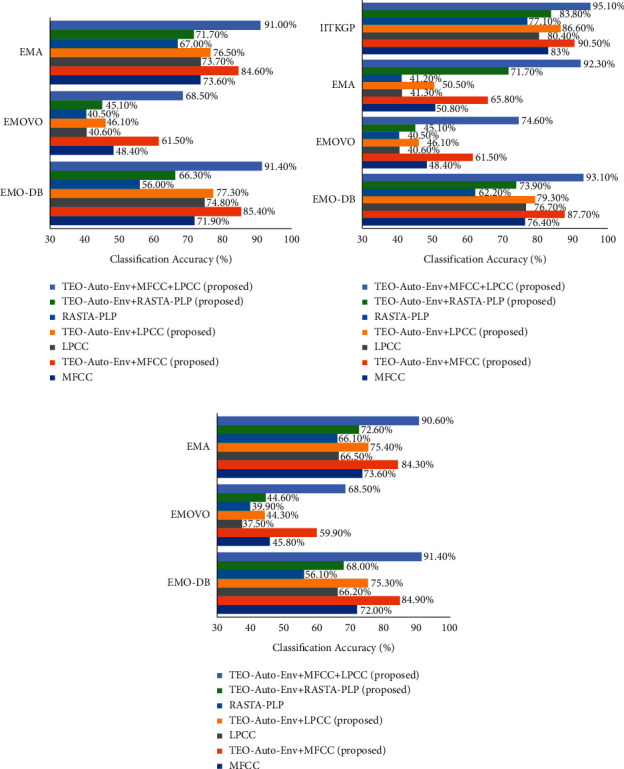
Comparison of the classification accuracies of the SSER system using the individual spectral feature and proposed feature extraction techniques of EMO-DB, EMOVO, IITKGP, and EMA database for (a) GD-male, (b) GD-female, and (c) SI case.

The SSER system is developed for four databases, namely, EMO-DB, EMOVO, IITKGP, and EMA for gender-dependent (GD) and speaker-independent (SI) cases. The results of these analyses are shown in Figures 7–10.

From the results depicted in Figures 7–9, it is clear that the classification accuracies of the stressed emotions are increased when the spectral features are combined with the TEO-Auto-Env feature for both GD and SI cases. Among all the combinations, TEO-Auto-Env feature when combined with MFCC & LPCC gave highest classification accuracy for all emotions compared to the rest of the feature extraction techniques.

From Figure 7, the stressed emotions with TEO-Auto-Env + MFCC + LPCC feature extraction technique are detected with improved accuracy of 94.2% (GD-male), 94.5% (GD-female), and 93.8% (SI) for anger; 90.2% (GD-male), 90.7% (GD-female), and 89.2% (SI) for fear; 91.9% (GD-male), 93.4% (GD-female), and 91.5% (SI) for boredom; 90% (GD-male), 94% (GD-female), and 92.1% (SI) for disgust; and 89.1% (GD-male), 93% (GD-female), and 90.6% (SI) for sad emotions of EMO-DB.

Only female speech of IITKGP database is considered in this work; hence, only GD-female case is considered. From Figure 6, the stressed emotion anger with TEO-Auto-Env + MFCC + LPCC feature extraction technique is detected with an accuracy of 95%.

From Figure 8, the stressed emotions with TEO-Auto-Env + MFCC + LPCC feature extraction technique are detected with improved accuracy of 78.6% (GD-male), 88.3% (GD-female), and 77.8% (SI) for anger; 66.4% (GD-male), 69.3% (GD-female), and 64.4% (SI) for fear; 67.5% (GD-male), 74.6% (GD-female), and 67.7% (SI) for disgust; and 67.4% (GD-male), 72.5% (GD-female), and 67% (SI) for sad emotions of EMOVO.

From Figure 9, the stressed emotions with TEO-Auto-Env + MFCC + LPCC feature extraction technique are detected with improved accuracy with 93.1% (GD-male), 92.3% (GD-female), and 91.8% (SI) for anger; and 90.6% (GD-male), 92.6% (GD-female), and 90.4% (SI) for sad emotions of EMA.

From Figure 10, it is clear that the SSER system developed specifically to recognize the stressed emotions can also recognize the other emotions efficiently. Hence, by using the proposed TEO-Auto-Env + MFCC + LPCC feature fusion technique in the SSER system, the highest classification accuracy is obtained as 91.4% (GD-male), 93.1% (GD-female), and 91.4% (SI) cases for EMO-DB data; 68.5% (GD-male), 74.6% (GD-female), and 68.5% (SI) cases for EMOVO data; 91% (GD-male), 92.3% (GD-female), and 90.6% (SI) cases for EMA data; and 95.1% (GD-female) case for IITKGP data.

### 4.1. Comparison of the Classification Accuracy of the Proposed SSER System Using TEO-Auto-Env + MFCC + LPCC Feature Extraction Technique with Previous Related Work for EMO-DB Data

In [11], the weighted spectral local “Hu” (HuWSF) moments were proposed for feature extraction of SER. HuWSF are commonly used in image feature extraction, whereas in speech, the first absolute orthogonal invariant of Hu moments is utilised for speech feature description, as these can find the energy concentrated to the center of energy gravity of 2D data based on the degree of evaluation. The SER system build with these HuWSF features provided a weighted average recall or classification accuracy of 74.71%, and later, these were combined with prosodic (PROS), zero crossing with peak amplitudes (ZCPA), LPCC, and MFCC to acquire an accuracy of 81.74% for all the emotions of EMO-DB database, whereas, by using the proposed method i.e., TEO-Auto-Env combined with MFCC and LPCC, the SSER system provided higher accuracy of 91.4% for EMO-DB data.

In [10, 12, 29], only six emotions of EMO-DB are considered apart from disgust emotion. In [10], the harmony features with combination of standard (qualitative), voice quality (VQ), and pitch interval (INT) features were used for SER and provided overall accuracy of 71.01%. In [29], discriminative band wavelet packet power coefficient (db-WPPC) features are used for SER, which provided an accuracy of 75.51%. These accuracies were less compared to the proposed SSER system i.e., 90.95%. In [12], Fourier parameters are used for feature extraction in SER. These parameters gave high accuracy in the detection of all the emotions of EMO-DB except for boredom emotion with 71.48% accuracy, whereas, in this proposed SSER system, the boredom emotion is detected with 91.5% accuracy.

In [13], a bioinspired ANFIS technique combined with MLP is used for SER, which gave an accuracy of 67.5% for anger and 52.5% for happiness emotions, and in [14], the correlation between glottal and auditory features of speech was considered, and based upon this, a GCZCMT feature is proposed for SER, which gave an accuracy of 83.25% for anger, 75.28% for happy emotions, and 86.96% for neutral, whereas the SSER system proposed using TEO-Auto-Env + MFCC + LPCC in this paper gave highest accuracy compared to these features with 93.8% for anger, 91.6% for happy emotions, and 89% for neutral.

From these comparisons, it is clear that the SSER system using the proposed feature fusion of TEO-Auto-Env combined with MFCC and LPCC gave the highest accuracy among all the other features.

## 5. Conclusion

The SSER system developed based on the feature fusion of the TEO-Auto-Env and Spectral features using k-NN classifier provided improved accuracy in case of all the databases compared to the SSER system build using individual spectral features. TEO-Auto-Env feature is based on TEO, as it is basically designed to improve the energies of the stressed emotions. Because of this reason, when TEO-Auto-Env is combined with spectral features, the features that were not able to be extracted from the stressed emotions were extracted, and the combination of the TEO-based feature with spectral features yielded better performance. SSER system is developed for four databases, namely, EMO-DB, EMOVO, IITKGP, and EMA for gender-dependent (GD) and speaker-independent (SI) cases. Among all the combinations, the SSER system developed using the feature fusion of TEO-Auto-Env MFCC and LPCC gave the highest accuracy in the classification of stressed emotions with 91.4% (SI), 91.4% (GD-male), and 93.1% (GD-female) for EMO-DB; 68.5% (SI), 68.5% (GD-male), and 74.6% (GD-female) for EMOVO; 90.6%(SI), 91% (GD-male), and 92.3% (GD-female) for EMA; and 95.1% (GD-female) for IITKGP female database compared to other feature fusions, which shows a favorable recognition performance in independent emotion speech recognition experiment. Also, the classification accuracy of the SSER system with the proposed feature showed higher accuracy compared to the features discussed in the literature.

## Figures and Tables

**Table 1:** Emo-DB database distribution.

	A	B	D	F	H	S	N
Number of speech samples	127	81	45	70	61	62	79

**Table 2:** EMOVO database distribution.

	D	F	A	J	Su	S	N
Number of speech samples	84	84	84	84	84	84	84

**Table 3:** IITKGP Telugu database distribution.

	Anger	Compassion	Happiness	Neutral
Number of speech samples	50	50	50	50

**Table 4:** EMA database distribution.

	Anger	Happiness	Sad	Neutral
Number of speech samples	170	170	170	170

## Data Availability

The datasets used and/or analyzed during the current study are available from the corresponding author on reasonable request.
